# Routing valley exciton emission of a WS_2_ monolayer via delocalized Bloch modes of in-plane inversion-symmetry-broken photonic crystal slabs

**DOI:** 10.1038/s41377-020-00387-4

**Published:** 2020-08-21

**Authors:** Jiajun Wang, Han Li, Yating Ma, Maoxiong Zhao, Wenzhe Liu, Bo Wang, Shiwei Wu, Xiaohan Liu, Lei Shi, Tian Jiang, Jian Zi

**Affiliations:** 1 State Key Laboratory of Surface Physics, Key Laboratory of Micro- and Nano-Photonics Structures (Ministry of Education) and Department of Physics, Fudan University, 200433 Shanghai, China; 2 College of Advanced Interdisciplinary Studies, National University of Defense Technology, 410073 Changsha, China; 3 Collaborative Innovation Center of Advanced Microstructures, Nanjing University, 210093 Nanjing, China

**Keywords:** Photonic crystals, Nanophotonics and plasmonics

## Abstract

The valleys of two-dimensional transition metal dichalcogenides (TMDCs) offer a new degree of freedom for information processing. To take advantage of this valley degree of freedom, on the one hand, it is feasible to control valleys by utilizing different external stimuli, such as optical and electric fields. On the other hand, nanostructures are also used to separate the valleys by near-field coupling. However, for both of the above methods, either the required low-temperature environment or low degree of coherence properties limit their further applications. Here, we demonstrate that all-dielectric photonic crystal (PhC) slabs without in-plane inversion symmetry (C_2_ symmetry) can separate and route valley exciton emission of a WS_2_ monolayer at room temperature. Coupling with circularly polarized photonic Bloch modes of such PhC slabs, valley photons emitted by a WS_2_ monolayer are routed directionally and are efficiently separated in the far field. In addition, far-field emissions are directionally enhanced and have long-distance spatial coherence properties.

## Separating valleys through photonic crystals

A method for separating novel excitonic valley states has been developed by researchers in China using an asymmetric photonic crystal. The emerging field of valleytronics aims to encode information using local minima (valleys) in the electronic band structure of certain materials, especially ultrathin layers of transition metal dichalcogenides (TMDCs). However, it is difficult to separate valley states at any distance from a sample, except at extremely low temperatures. To address this problem, Lei Shi at Fudan University in Shanghai and co-workers deposited a TMDC layer on a photonic crystal, which was etched with triangular holes to break its natural symmetry. The photonic crystal’s circularly polarized states interact with the exciton valleys, so that photons from different valley states could be separated far from the sample, even at room temperature.

## Introduction

**Fig. 1:**
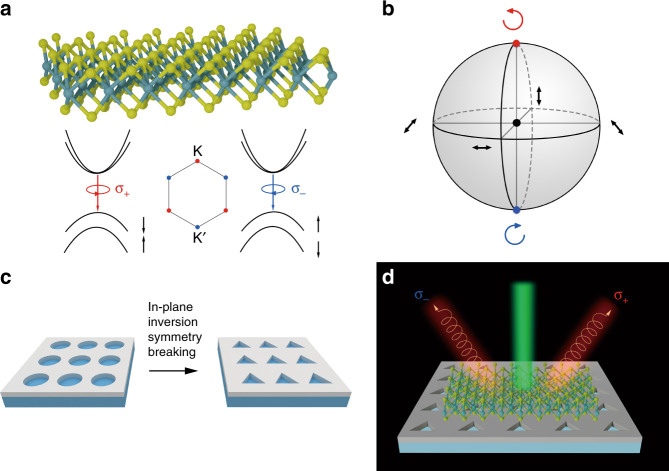
Schematics and principle. **a** Schematic of the WS_2_ monolayer and optical selection rules at the K and K’ valleys. σ_+_ (σ_−_) excitation corresponds to interband optical transition at the K (K’) valley. **b** The normalized Poincaré sphere. Different azimuthal positions on the equator correspond to different linearly polarized states. Two poles correspond to two types of circularly polarized states. The centre of the Poincaré sphere corresponds to vortex singularity. **c** PhC slabs with C_4_ symmetry and without in-plane inversion symmetry. **d** Illustration of the photoluminescence of the WS_2_ monolayer on the PhC slab without in-plane inversion symmetry. Valley photons with different chiralities separate and radiate directionally in the far field

The emergence of two-dimensional transition metal dichalcogenides (TMDCs) has attracted tremendous interest for their possible applications in valleytronics^1–12^. Due to the broken inversion symmetry in TMDCs, two types of degenerate yet inequivalent valleys (labelled K and K’ valleys) appear at the corners of the first Brillouin zone, as shown in Fig. 1a. Interband transitions at valleys, which are excitonic transitions in nature for TMDCs, show highly valley-dependent optical selection rules^4–6,13–18^. This controllable selective population of certain valleys, called valley polarization, offers a new valley degree of freedom, spawning the emergent field of valleytronics.

To develop valleytronic devices based on TMDCs, effective approaches to separate valleys in the near or far field are indispensable. One feasible way is to selectively excite valleys by utilizing different external stimuli such as optical and electric fields^14–18^, while the usually required low-temperature environment makes it difficult for practical applications. Due to the powerful ability of manipulating light, nanostructures^19–21^ are also proposed to separate valleys^22–32^. For example, based on either the transverse spin momentum of surface plasmons^27,28^ or the variable geometric phase of metasurfaces^31^, valley separation was reported to be achieved in the near or far field at room temperature. However, both the intrinsic loss of metal materials and the localized spatial distribution of resonant modes of nanoantennas limit efficient valley separation, leading to a low degree of valley polarization^24–30^. As a counterpart of metasurfaces, photonic crystals (PhCs) eliminate all these disadvantages due to delocalized photonic Bloch modes and low-intrinsic-loss dielectric constituents. In addition, these Bloch modes are found to have peculiar polarization properties. With these attractive properties, PhCs have been widely applied in various studies, such as bound states in the continuum^33–37^, topological valley photonics^38,39^, PhC lasers^40–42^, and spontaneous emission control of TMDCs^43^. However, to date, there are no reports of effective valley separation in TMDCs by using PhCs.

In this article, we demonstrate that two-dimensional all-dielectric PhC slabs without in-plane inversion symmetry can be used to efficiently separate valley exciton emission of a WS_2_ monolayer in the far field at room temperature. The valley exciton emission is routed with high directionality and a high degree of valley polarization, as shown in Fig. 1d. For this type of PhC slab, paired delocalized Bloch modes with different circular polarizations not only play a critical role in separating and enhancing directional valley exciton emission but also lead to spatial coherence properties of the emission field, which have not been discussed in past studies. Experimentally, the angle-resolved photoluminescence (PL) results directly show efficient valley separation in the far field, with a degree of valley polarization up to 88%. Time-resolved PL measurements indicate a 75% enhancement of the exciton radiative rate. In addition, the double-slit interference results reveal that the spatial coherence length of the emission field for a WS_2_ monolayer on a PhC slab without in-plane inversion symmetry is longer than 6 microns (29 microns in theory).

### Principle of separating and routing valley exciton emission

Analogous to electronic band structures in solids, Bloch scattering by periodic artificial atoms of PhC slabs alters the dispersion relation of light in the slab, resulting in photonic bands^44^. Each optical state in photonic bands corresponds to a delocalized Bloch mode with well-defined energy and momentum. Modes above the light cone are radiative due to coupling to the free space^44^. For these radiative modes, their polarization states in the far field are strictly defined. The corresponding polarization states of radiative Bloch modes in an arbitrary photonic band can be further projected into the structure plane and mapped onto the Brillouin zone, defining a polarization field in the momentum space^33,34^. These polarization properties in principle could be used to control the radiation of luminescent materials. However, owing to high rotation symmetry, the polarization field is nearly linear in most PhC slabs^45^. As a consequence, the polarization states of those PhC slabs can only cover a belt near the equator of the Poincaré sphere^46^ (a space to describe all polarization states, shown in Fig. 1b). With a large area including two poles not covered, it is useless for us to utilize these Bloch modes of PhC slabs to separate valley exciton emission of TMDCs.

In contrast, as is known, broken inversion symmetry is of vital importance in the appearance of inequivalent valley excitons in TMDCs. Similarly, we recently reported that paired circularly polarized states with different chiralities emerge from vortex singularities after breaking the in-plane inversion symmetry of PhC slabs^46^, as shown in Fig. 1c. Then, in addition to the areas near the equator, the polarization states cover the whole sphere, including two poles of the Poincaré sphere, corresponding to polarization states with a high degree of circular polarization in momentum space. Therefore, this type of PhC slab with circularly polarized radiative states could be an ideal platform for us to separate valley exciton emission of TMDCs in the far field, as illustrated in Fig. 1d.

First, valley photons could couple to circularly polarized states with corresponding chirality and become separated in the momentum space. Second, these Bloch modes are delocalized and could be used in coherent emission^47,48^. The spatial coherence properties of the emission field lay the foundation for the directionality and highly efficient separation of valley exciton emission of a WS_2_ monolayer. A detailed discussion is provided in Supplementary Material section 1.

## Results and discussion

**Fig. 2:**
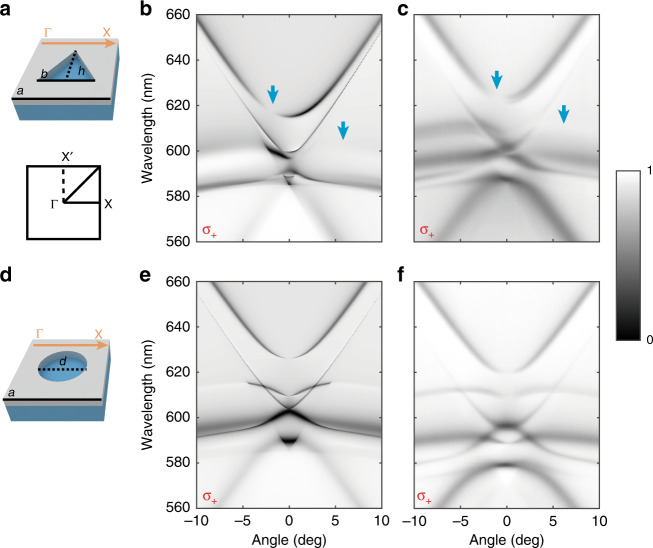
Simulated and experimentally measured angle-resolved transmittance spectra. **a** Unit cell of the photonic crystal slab without in-plane inversion symmetry. Triangular air holes are etched in the Si_3_N_4_ layer. **b**, **c** Simulated and measured angle-resolved transmittance spectra in the visible range under σ_+_-polarized incidence. The incidence plane is along the Γ-X direction. Diminished regions indicated by blue arrows imply the existence of circularly polarized states after breaking the in-plane inversion symmetry. **d** Unit cell of photonic crystal slabs with in-plane inversion symmetry. Circular air holes are etched in the Si_3_N_4_ layer. **e**, **f** Simulated and measured angle-resolved transmittance spectra in the visible range under σ_+_-polarized incidence. The spectra are symmetric for the PhC slab with in-plane inversion symmetry

To demonstrate the existence of opposite circularly polarized states in momentum space, we designed an in-plane inversion-symmetry-broken PhC slab and studied the transmittance spectra in theory and experiment, as shown in Fig. 2. The slabs here are made of silicon nitride (Si_3_N_4_, refractive index ∼2) and silicon dioxide (SiO_2_, refractive index ∼1.5). The thickness of the Si_3_N_4_ layer is 150 nm. The thickness of the SiO_2_ layer is 500 microns, which could be considered infinite compared to the wavelength of visible light. Square lattices of holes with a period a = 390 nm are etched in the Si_3_N_4_ layer. To break the in-plane inversion symmetry, the shape of the etched hole in a unit cell is set as an isosceles triangle, with the height h and baseline length b of the triangle being equal (h = d = 250 nm), as shown in Fig. 2a. More details about the sample design can be found in Supplementary Material section 3.

We first simulated the angle-resolved transmittance spectra under σ_+_-polarized incidence by Rigorous Coupled Wave Analysis (RCWA), with the incidence plane along the Γ-X direction. The spectra are asymmetric, and there are some diminished regions on the photonic bands, indicated by blue arrows in Fig. 2b. These diminished regions correspond to the nonexcited states under σ_+_-polarized incidence. Hence, those states in the diminished regions are σ_−_ polarized. Changing the incident light to σ_−_ polarization, the diminished regions switch to the other side (Fig. S1b). To show this effect experimentally, we fabricated samples using electron-beam lithography and reactive ion etching (for more details, see Methods). By using a homemade polarization-resolved momentum-space imaging spectroscopy system (Fig. S4), angle-resolved transmittance spectra are measured (Fig. 2c), in accordance with the simulation. Both the simulated and experimentally measured results confirmed the appearance of optical modes with a high degree of circular polarization in our designed PhC slab. For comparison, we also researched the angle-resolved transmittance spectra of the PhC slab with in-plane inversion symmetry. Shown in Fig. 2d, the designed shape of the etched hole in the unit is a circle (diameter d = 210 nm). As expected, we did not observe asymmetric spectra under σ_+_-polarized incidence in either the simulation or experiment, as shown in Fig. 2e, f. When changing the incidence to σ_−_ polarization, the transmittance spectra are the same as those in the case of σ_+_ polarization (Fig. S1c). The results demonstrate that by breaking the in-plane inversion symmetry of PhC slabs, circularly polarized states emerge in photonic bands.

**Fig. 3:**
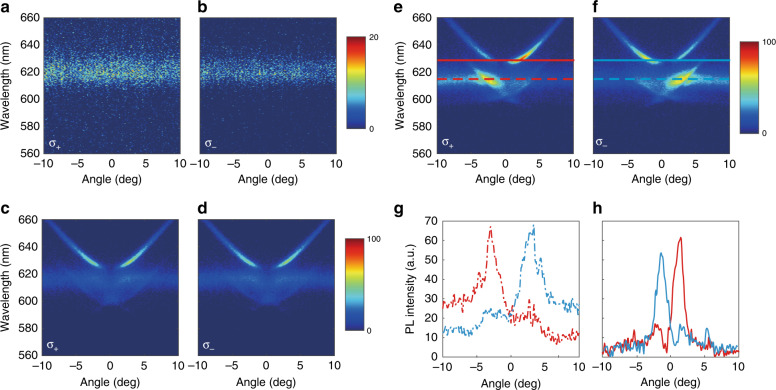
Angle-resolved PL spectra of the WS_2_ monolayer on different substrates at room temperature. **a**–**f** Angle-resolved PL spectra of the WS_2_ monolayer on three different substrates with σ_+_ (σ_−_) detection along the Γ-X direction. **a** and **b** correspond to the WS_2_ monolayer on a flat substrate. **c** and **d** correspond to the WS_2_ monolayer on the PhC slab with in-plane inversion symmetry. **e** and **f** correspond to the WS_2_ monolayer on the PhC slab without in-plane inversion symmetry. **g**, **h** Separation of σ_+_ (red) and σ_−_ (blue) polarized light at 615 nm (dotted line) and 628 nm (solid line) in **e**, **f**. The maximum calculated *P* reaches 84%

The large area of the WS_2_ monolayer is grown on a Si/SiO_2_ substrate by the CVD process and then transferred onto PhC slabs. Both PhC slabs and part of the unstructured flat Si_3_N_4_ substrate are covered (Fig. S10). To study the PL distribution in the far field, angle-resolved PL spectra are measured (Supplementary Material section 5), as shown in Fig. 3a–f. The detection plane is along the Γ-X direction, in accordance with the transmittance spectra measurement in Fig. 2. We selected σ_+_ (σ_−_) PL by placing a quarter-wave plate and a linear polarizer in the detection path (Fig. S4). Figure 3e, f shows the asymmetric σ_+_ (σ_−_) PL spectra of the WS_2_ monolayer on the PhC slab without in-plane inversion symmetry. The σ_+_ (σ_−_) PL enhanced regions correspond to regions with a high degree of σ_+_ (σ_−_) polarization in photonic bands. Figure 3a, b shows σ_+_ (σ_−_) PL spectra of the WS_2_ monolayer on a flat substrate. Figure 3c, d shows σ_+_ (σ_−_) PL spectra of the WS_2_ monolayer on the PhC slab with in-plane inversion symmetry. Different from those in Fig. 3e, f, all spectra in Fig. 3a–d are symmetric for both σ_+_ and σ_−_ detection. From the abovementioned experimental results, we can draw the conclusion that, as shown in the asymmetric spectra, valley photons emitted by the WS_2_ monolayer have been separated in the far field by PhC slabs without in-plane inversion symmetry. In addition, we performed time-resolved PL measurements at room temperature (Supplementary Material section 10). Compared with that for the WS_2_ monolayer on a flat substrate, the exciton radiative rate, namely, the reciprocal of radiative lifetime, is enhanced by 75% when the WS_2_ monolayer is on a PhC slab without in-plane inversion symmetry.

To further study the degree of separation in Fig. 3e, f, we plotted the angle-resolved σ_+_ (σ_−_) PL spectra for a single wavelength, as shown in Fig. 3g, h. The dotted line refers to 615 nm, and the solid line refers to 628 nm, which are also marked in Fig. 3e, f. We observed that σ_+_ (red) and σ_−_ (blue) PL maximums separately appear at different angles. The σ_+_ and σ_−_ PL peaks are separated by nearly 6 degrees at 615 nm and 3 degrees at 628 nm. For comparison, PL spectra on a PhC with in-plane inversion symmetry for corresponding wavelengths are shown in Fig. S5, with the σ_+_ and σ_−_ PL maximums overlapping at the same angle. We also show that the photoluminescence of the WS_2_ monolayer on this PhC slab without in-plane inversion symmetry is highly directional. As shown in Fig. 3g, h, the full width at half maximum of the PL peaks (∆*θ*) is less than 3 degrees at 615 nm and 2 degrees at 628 nm. This result is due to the delocalized property of Bloch modes, leading to the long-distance spatial coherence property of the far-field emission by the WS_2_ monolayer on PhC slabs. According to the Fourier relation between momentum and position, a wide distribution in the real space means that the mode is localized inside a small area in the momentum space. This effect corresponds to the small angle distribution of the far-field emission, i.e., the directional emission, and will be further discussed later in this article. For this reason, although the separation of σ_+_ and σ_−_ PL peaks is small, the valley exciton emission could still be efficiently separated in the far field. Further, we quantify the degree of valley polarization by

$$P\left( \theta \right) = \frac{{I_ + \left( \theta \right) - I_ - (\theta )}}{{I_ + \left( \theta \right) + I_ - (\theta )}}$$

where *I*_*+*_ (*I*_*−*_) refers to the PL intensity with σ_+_ (σ_−_) polarization for a single wavelength, and *θ* is the radiation angle. The degree of valley polarization is plotted in Fig. S7, with the maximum degree of valley polarization calculated up to 84%. These results indicate that the PL of the WS_2_ monolayer on the PhC slab without in-plane inversion symmetry is highly directional and has a high degree of valley polarization.

**Fig. 4:**
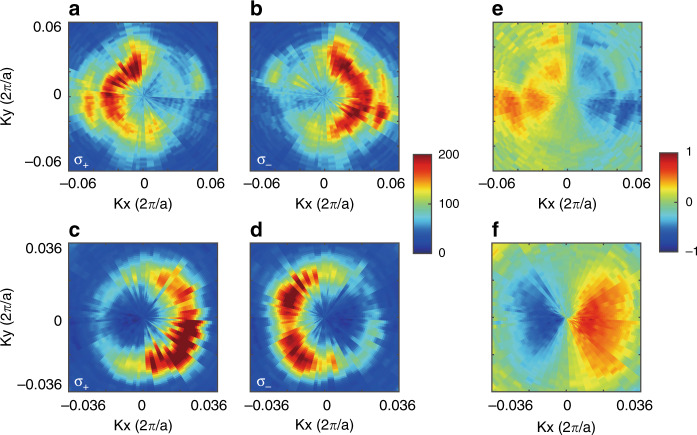
Experimental measurement of PL spectra and valley polarization in momentum space. **a**–**d** σ_+_ and σ_−_ PL intensity distribution in momentum space at 615 nm (upper) and 628 nm (lower). These are for the WS_2_ monolayer on the PhC slab without in-plane inversion symmetry. **e**, **f** Images of valley polarization *P*(*k*) in momentum space. The maximum calculated *P* reaches 88%

Based on the measured angle-resolved σ_+_ (σ_−_) PL spectra of the WS_2_ monolayer on the PhC slab without in-plane inversion symmetry, we mapped the PL intensity distribution of a single wavelength in momentum space, as shown in Fig. 4a–d. The upper (lower) row corresponds to 615 (628) nm. The PL spectra along different directions in momentum space were measured by rotating the sample in-plane relative to the entrance slit of the imaging spectrometer. The projected momentum *k* is calculated by *k* = *k*_*0*_sin*θ* (*k*_*0*_ = 2π*/λ* is the wavevector of light in the free space, *θ* is the emission angle relative to normal of the sample plane). The intensity distribution of σ_+_ (σ_−_) PL in momentum space confirmed that the PhC slab without in-plane inversion symmetry leads to directional valley exciton emission.

Then, we used *P*(*k*) to qualify the degree of valley polarization in momentum space, which is similarly defined by $P\left( k \right) = \frac{{I_ + \left( k \right) - I_ - (k)}}{{I_ + \left( k \right) + I_ - (k)}}$, as shown in Fig. 4e, f. Here, *I*_*+*_ (*I*_*−*_) refers to the PL intensity with σ_+_ (σ_−_) polarization for a single wavelength. Experimentally, the maximum calculated *P* reaches 88%, as shown in Fig. 4f. Note that the maximum *P* did not appear along the Γ-X direction in momentum space. The result is as expected because the circular polarized states of the designed PhC slab without in-plane inversion symmetry are slightly shifted from the Γ-X direction in momentum space^40^. The sign of *P*(*k*) reverses at opposite sides of the momentum space, demonstrating the separation of valley exciton emission with different chiralities. In contrast, we also measured and calculated *P*(*k*) of the emission by a WS_2_ monolayer placed on a flat substrate, and *P*(*k*) was negligible (Fig. S12).

**Fig. 5:**
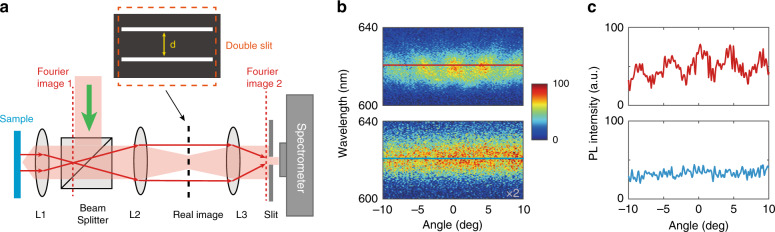
Experimental results of Young’s double-slit interference. **a** Schematic view of the experimental setup. L, Lens. The double-slit distance is d. The double slit is mounted on the real image plane. The red arrow lines show the radiation fields from two different positions on the sample. **b** The experimental results for the case of a 6-micron effective double-slit distance. The real double-slit distance d is 120 microns. The magnification of the real image is 20, so the effective double-slit distance on the sample is 6 microns. The upper panel shows the WS_2_ monolayer on the PhC slab without in-plane inversion symmetry. The lower panel is for the WS_2_ monolayer on a flat substrate, with the signal intensity shown at two-fold magnification. The detection plane is along the Γ-X direction. **c** The interference intensity distribution in **b** at 621 nm. The far-field emission of the WS_2_ monolayer on the PhC slab without in-plane inversion symmetry has a long-distance spatial coherence property

In addition to valley-related directional emission in momentum space, we expected the spatial coherence property of emission by the WS_2_ monolayer on the PhC slab without in-plane inversion symmetry. Young’s double-slit experiments were performed, as shown in Fig. 5. The experimental setup is illustrated in Fig. 5a, and the working principle is based on Fourier transformation. The double slit is mounted on the real image plane inside the optical measurement setup to select radiation fields from two different positions on the sample. The radiation fields from these two positions intersect with each other on Fourier image 2 at the entrance of the spectrometer. Therefore, the spatial coherence properties on the surface of the sample could be directly detected in the far field. Changing the etched depth of the PhC slab, we were able to overlap the measured photonic band with the PL spectra of the WS_2_ monolayer to obtain enough signal intensity. Interference fringes are observed in the angle-resolved PL spectra along the Γ-X direction, as shown in Fig. 5b. The red-marked line is further plotted in Fig. 5c, showing the interference intensity distribution at 621 nm. The fringe visibility *V* is calculated to be ~50%, defined by $V = \frac{{I_{{\mathrm{max}}} - I_{{\mathrm{min}}}}}{{I_{{\mathrm{max}}} + I_{{\mathrm{min}}}}}$, where *I*_max_ and *I*_min_ are the intensities of adjacent maximums and minimums^49^. In this measurement, the real double-slit distance d is 120 microns. The scanning electron microscopy image of the double slit is presented in Fig. S13. The magnification of the real image is 20, so the effective double-slit distance on the sample is 6 microns. The 6-micron effective double-slit distance is almost ten times the emission wavelength, demonstrating that the measured spatial coherence length is larger than 6 microns. Moreover, the spatial coherence length could be calculated by $\frac{\lambda }{{{\Delta}\theta }}$ in theory, which is widely used in optical coherence theory^50^. Here, ∆*θ* is ~0.0215 (1.23 degrees) at 621 nm (Fig. S14), and the calculated spatial coherence length is ~29 microns. In comparison, no interference fringes are observed when the WS_2_ monolayer is placed on a flat substrate, as shown in Fig. 5b, c. This result means that the far-field emission of the WS_2_ monolayer on a flat substrate has no long-distance spatial coherence property. Hence, we reveal that the far-field emission by the WS_2_ monolayer on the PhC slab without in-plane inversion symmetry has a long-distance spatial coherence property. This property of the PhC slab extends the coherence control on the PL of the WS_2_ monolayer from temporal coherence to spatial coherence.

In summary, we proposed in-plane inversion-symmetry-broken all-dielectric photonic crystal slabs to route valley exciton emission of a WS_2_ monolayer in the far field at room temperature. By breaking the in-plane inversion symmetry of the PhC slab, we observed paired circularly polarized states with different chiralities emerge from vortex singularities. Via coupling with those delocalized Bloch modes, valley photons emitted by the WS_2_ monolayer were separated in momentum space, and the exciton radiative rate was significantly enhanced. In addition, both the directional emission and the long-distance spatial coherence property benefit the applications of in-plane inversion-symmetry-broken PhC slabs to route valley exciton emission. In addition, our method could be extended to manipulate valley exciton emission of other TMDC monolayers. The ability of these PhC slabs to transport valley information from the near field to the far field would help to develop photonic devices based on valleytronics.

## Methods

### Sample fabrication

#### The fabrication of a photonic crystal slab

The sample structure was two slab layers, with a thin silicon nitride layer on the silicon dioxide substrate. The silicon dioxide substrate was cut from a 500-micron-thick quartz wafer. Then, a silicon nitride layer was grown on a silicon dioxide substrate by plasma-enhanced chemical vapour deposition (PECVD). The thickness of the grown silicon nitride layer was nearly 150 nm, and the thickness could be tuned by controlling the deposition time. To fabricate the designed structure, the raw sample was spin-coated with a layer of positive electron-beam resist (PMMA950K A4) and an additional layer of conductive polymer (AR-PC 5090.02). Then, a hole array mask pattern was fabricated onto the PMMA layer using electron-beam lithography (ZEISS sigma 300). The sample was further processed by reactive ion etching (RIE). Anisotropic etching was achieved by RIE using CHF_3_ and O_2_. The patterned PMMA layer acted as a mask and was eventually removed by RIE using O_2_. The size of every designed structure is ~80 × 80 microns.

#### Transfer process for the WS_2_ monolayer

The CVD WS_2_ monolayer on the Si/SiO_2_ substrate was spin-coated with poly(L-lactic acid) (PLLA) before baking for 5 minutes at 70 °C. Afterwards, a PDMS elastomer was placed on top of the PLLA film and then torn off. The composite was then attached to a glass slide and put under a microscope on a transfer stage. The PhC slab placed under the glass slide was aligned carefully using the microscope, and the glass slide was lowered to contact the PhC slab. The stage was heated to 70 °C to improve the adhesion, and then, the glass slide was lifted with PDMS, leaving a WS_2_ monolayer on the PhC slabs. After dissolving PLLA in dichloromethane, the WS_2_ monolayer was finally transferred to the designed photonic crystal slabs.

### Optical measurements

#### Experimental measurements of time-resolved PL

Please see Supplementary Material section 4 for the schematics and discussions.

#### Measurement setup of the polarization-resolved momentum-space imaging spectroscopy system and double-slit experiment

Please see Supplementary Material section 5 for the schematics and discussions.

### Simulations

The transmittance spectra were simulated by Rigorous Coupled Wave Analysis (RCWA). The periodic boundary conditions were applied in the x and y directions. The polarization angle was set to π/4, and the phase difference was set to π/2 or 3π/2 to obtain circularly polarized incidence (the polarization angle 0 (π/2) corresponds to p(s) polarization). The Si_3_N_4_ refractive index was set to 2, and the SiO_2_ refractive index was set to 1.5. All the materials were considered to have no loss in visible light.

## Supplementary information

supplementary materials

## Data Availability

The data that support the findings of this study are available from the authors on reasonable request; see the author contributions for specific data sets.
