# Supplementary material for: Routing valley exciton emission of a WS2 monolayer via delocalized Bloch modes of in-plane inversion-symmetry-broken photonic crystal slabs
Source: Light Sci Appl. 2020 Aug 21;9:148. doi: 10.1038/s41377-020-00387-4 (PMC7442784; doi:10.1038/s41377-020-00387-4)
Supplement: Supplementary file 1 — supplementary materials [file 41377_2020_387_MOESM1_ESM.docx]

Supplementary Information: Routing valley exciton emission of a WS_2_ monolayer via delocalized Bloch modes of in-plane inversion-symmetry broken photonic crystal slabs

Jiajun Wang^1,*^, Han Li^2,*^, Yating Ma^2^, Maoxiong Zhao^1^, Wenzhe Liu^1^, Bo Wang^1^,

Shiwei Wu^1,3^, Xiaohan Liu^1,3^, Lei Shi^1,3,†^, Tian Jiang^2,‡^, and Jian Zi^1,3,§^

^1^ State Key Laboratory of Surface Physics, Key Laboratory of Micro- and Nano-Photonics Structures (Ministry of Education) and Department of Physics, Fudan University, Shanghai 200433, China

^2^ College of Advanced Interdisciplinary Studies, National University of Defense Technology, Changsha 410073, China

^3^ Collaborative Innovation Center of Advanced Microstructures, Nanjing University, Nanjing 210093, China

^*^ These authors contributed equally to this work.

^†^ lshi@fudan.edu.cn

^‡^ tjiang@nudt.edu.cn

^§^ jzi@fudan.edu.cn

**Content**

**1. Discussions about spatial coherence properties of the far-field emission by WS_2_ monolayer on all-dielectric PhC slabs**

**2. Angle-resolved transmittance spectra with σ_-_ polarized incidence**

**3. Illustration of structure parameters’ design**

**4. Schematic view of the lab-built PL system**

**5. Schematic view of polarization-resolved momentum-space imaging spectroscopy system**

**6.** **Angle-resolved PL spectra of WS_2_ monolayer on substrate with in-plane inversion symmetry**

**7. Directional PL enhancement in the far field**

**8. Calculated degree of valley polarization**

**9. Simulated transmittance along high-symmetry directions and Iso-frequency contours**

**10. Time-resolved PL measurement under linearly polarized pulsed laser excitation**

**11. Optical photos of monolayer WS_2_ on different substrates**

**12. Near-filed simulations and experimental PL images for the selective coupling of circularly polarized modes in the in-plane inversion-symmetry broken PhC slab**

**13. Degree of valley polarization in momentum space for WS_2_ monolayer on flat substrate**

**14.** **The scanning electron microscopy image of the double-slit**

**15.** **Angle-resolved spectra without circularly-polarized detection**

**16. Simulated angle-resolved transmittance spectra of PhC with up-down mirror symmetry about the centric sample plane**

**17. References**

**1. Discussions about spatial coherence properties of the far-field emission by WS_2_ monolayer on all-dielectric PhC slabs**

When we pump the WS_2_ monolayer placed on a substrate, different positions of the WS_2_ could be considered as point-source emitters, emitting photons separately. These emitters are uncorrelated except cases like lasing or strong coupling. When the WS_2_ monolayer is placed on the all-dielectric PhC slabs which support delocalized photonic Bloch modes, emitted photons will firstly couple to the photonic modes and then radiate to the free space. Due to the complex parallel wavevector of photonic modes, photons could propagate in the PhC slabs. The field at any position is contributed by all emitters rather than just the nearest one, hence the emission field has spatial coherence property^1,2^. In contrast, when the WS_2_ monolayer is placed on flat substrate which doesn’t support resonance modes, emitted photons by WS_2_ monolayer will radiate to the free space directly and randomly, and the emission field doesn’t have spatial coherence property.

The delocalized photonic modes of all-dielectric PhC slabs play a vital role in the spatial coherence property of the emission field. Because of their high photonic density of states inside a very thin slab, the spontaneous emission rate of the WS_2_ monolayer could be enhanced^3, 4^. And the delocalized modes contribute to emitted photons’ transverse propagation along slabs. Then the chosen non-loss all-dielectric materials reduce the dissipation during the propagation process. All these keep the long propagation length, enhancing the spatial coherence in the near-field region. Further, according to the Fourier relation between momentum and position, wide distribution in the real space means that the mode is localized inside a small area in the momentum space. We can predict the directional emission by WS_2_ monolayer on PhC slabs^2^.

The spatial coherence properties lay the foundation for high-efficient separation of valley excitons in a WS_2_ monolayer and the application to route the valley exciton emission. All these properties could be directly verified by angle-resolved spectroscopy combined with Young’s double-slit experiment, shown in our article. In **Section 12**, near-filed simulations are also offered to support our claims.

**2. Angle-resolved transmittance spectra with σ_-_ polarized incidence**


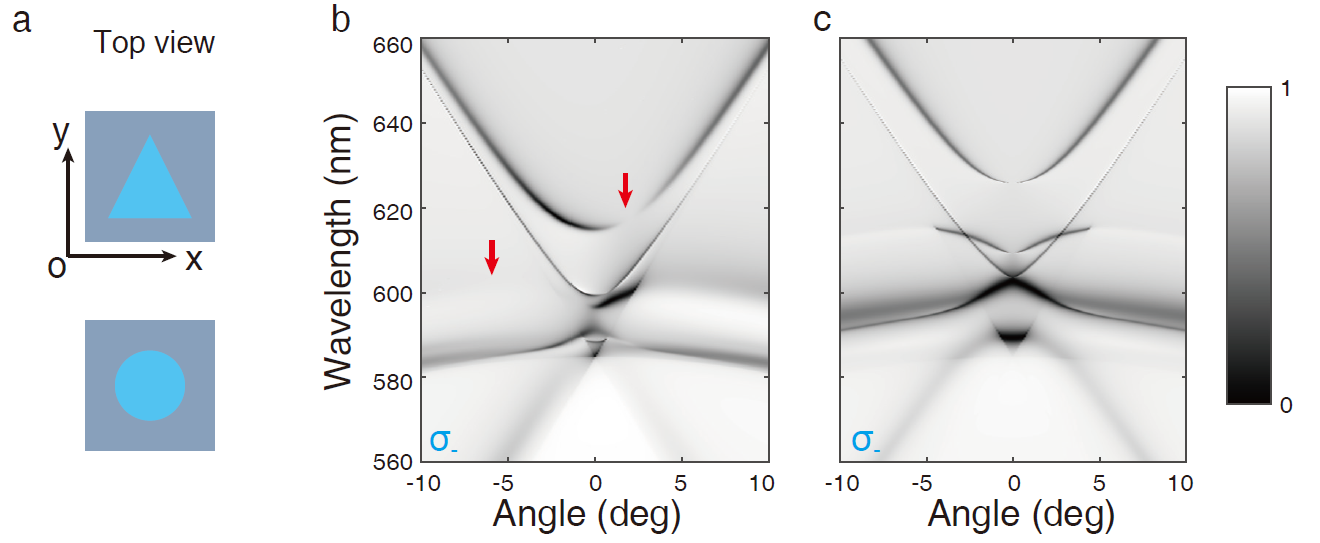


Figure S1: **Simulated angle-resolved transmittance spectra in the visible range under σ_-_ polarized incidence.** The incidence plane is along Γ-X direction. a. Top view of the unit cells of PhC slabs. A Cartesian frame is used to show the relative direction. The Γ-X direction is parallel to the x axis. b. Transmittance spectra of PhC slab without in-plane inversion symmetry. The spectra are asymmetric, and the diminished regions pointed out by red arrows correspond to σ_+_ polarized photonic states. (b) Transmittance spectra of PhC slab with in-plane inversion symmetry. The spectra are symmetric. The structure’s parameters are shown in Fig. 2.

**3. Illustration of structure parameters’ design**


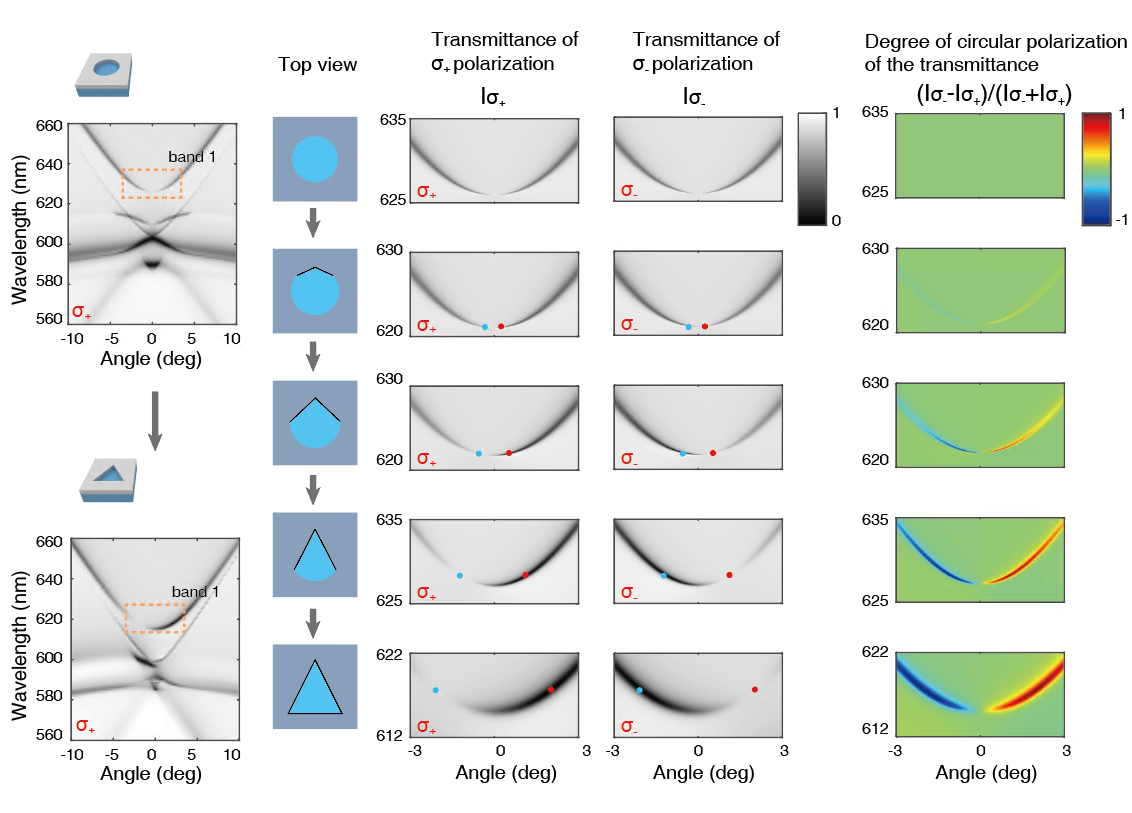


Figure S2: **Illustration of structure parameters’ design.** The angle-resolved transmittance spectra under circularly polarized incidence are along Γ-X direction, simulated by RCWA. The blue and red points marked in the transmittance spectra correspond to σ_-_ and σ_+_ polarized states.

We take a part of band 1 as an example to show the evolution of circularly polarized states with the varying parameters. The circular air holes are changed into triangular air holes by gradually breaking the in-plane inversion symmetry of the unit cell. In this process, we keep the mirror symmetry of the structure so that the circularly polarized states are also mirror-symmetric in the momentum space. Besides, the air holes’ area is also slightly changed to make sure the circularly polarized states emerge in the expected wavelength.

The blue and red points marked in the transmittance spectra correspond to σ_-_ and σ_+_ polarized states. And the circularly polarized Bloch states immediately emerge as soon as the in-plane inversion symmetry is broken. The more the in-plane inversion symmetry is broken, the farther σ_-_ and σ_+_ polarized states separate from each other in the far field. We also calculate the degree of circular polarization of the transmittance to characterize the circular polarization evolution of band 1. The overall degree of circular polarization of the band 1 increases when we gradually change the circular air holes into triangular air holes.

**4. Schematic view of t****he lab-built PL system**


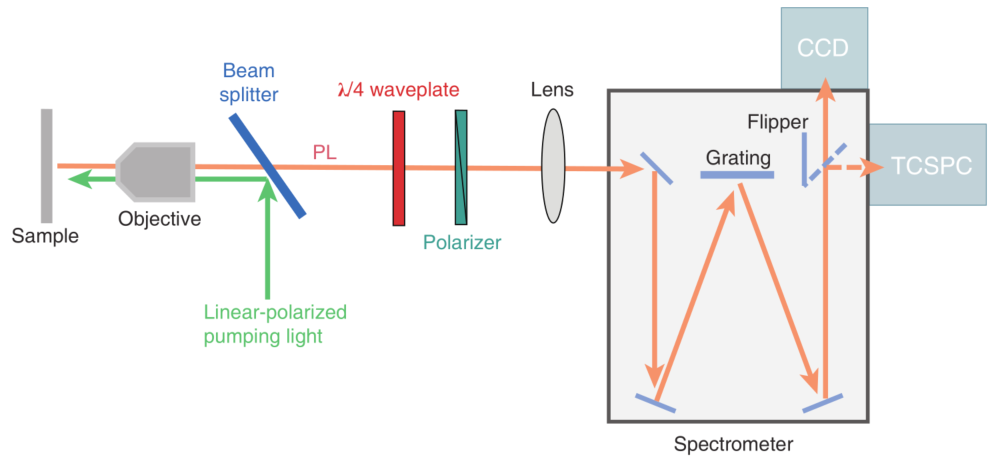


Figure S3: **Schematic view of the lab-built PL system.** TSPC: time-correlated single photon counting.

There are two main modules for this PL system. In the first module, by setting the grating to zero order, CCD in spectrometer can capture PL images in real space. Controlling the *λ*/4 waveplate and polarizer, we can select the PL components with different polarization. And the *λ*/4 waveplate and polarizer could be moved out of the system, then we can get the total PL information. In the second module, we can use the TCSPC module to perform time-resolved PL measurements. In the TR-PL experiment, pumping light is a 400 nm laser of 100 fs pulse-width. TR-PL decay traces are collected by a time-correlated single photon counting (TCSPC) device. After deconvolution of measured decay traces with instrument response function (IRF), TR-PL decay traces are fitted by a biexponential model consisting of non-radiative recombination and radiative recombination processes^5^.

**5. Schematic view of polarization-resolved momentum-space imaging spectroscopy system**


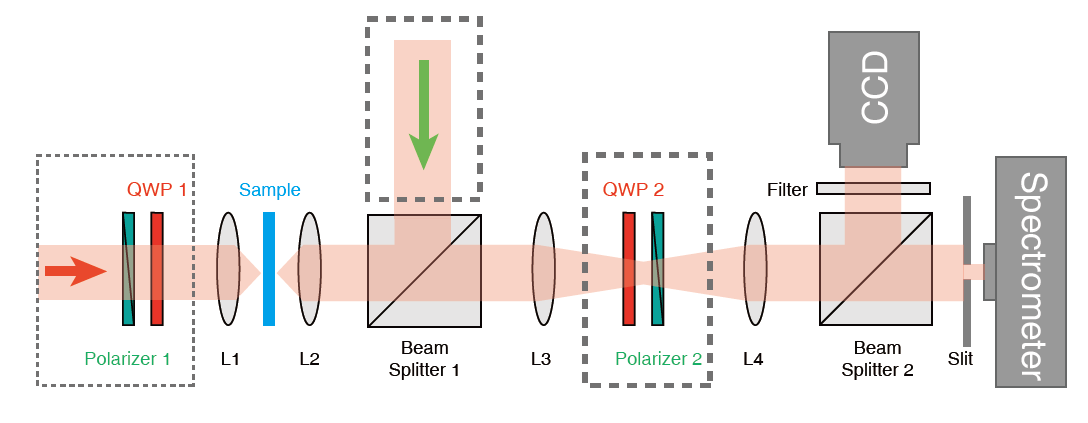


Figure S4: **Schematic view of the experimental setup.** L, Lens. QWP, quarter-wave plate.

The system is based on Fourier transformation. After light passing the objective lens (L2), the real space spectral information is transformed into momentum space. It has two working modes. In the first mode, by using a bandpass filter, we can image the iso-frequency contour in the entire first Brillouin zone (FBZ) onto a two-dimensional charge-coupled-device (CCD). In the second mode, we put a spectrometer in the plane conjugate to the sample plane. The photonic bands along different directions in the FBZ are observed by rotating the sample in plane relative to the entrance slit of the spectrometer^6^.

This system could be used in both transmittance spectra and photoluminescence measurement. When measuring the transmittance spectra, we use incoherent broadband light source (the red arrow). Linear polarizer 1 and QWP 1 are inserted between the source and lens 1. By controlling the relative angle of linear polarizer 1 and QWP 1, we can change the incident polarization to circularly polarized. Then we can get the angle-resolved transmittance spectra within the whole visible light wavelength range by single-shot. When measuring the photoluminescence, the source is the 532 nm laser (the red arrow). QWP 2 and Linear polarizer 2 are inserted between the lens 3 and lens 4. By controlling the relative angle of QWP 2 and linear polarizer 2, we can selectively detect the σ_+_ and σ_-_ polarized light.

**6.** **Angle-resolved PL spectra of WS_2_ monolayer on substrate with in-plane inversion symmetry**


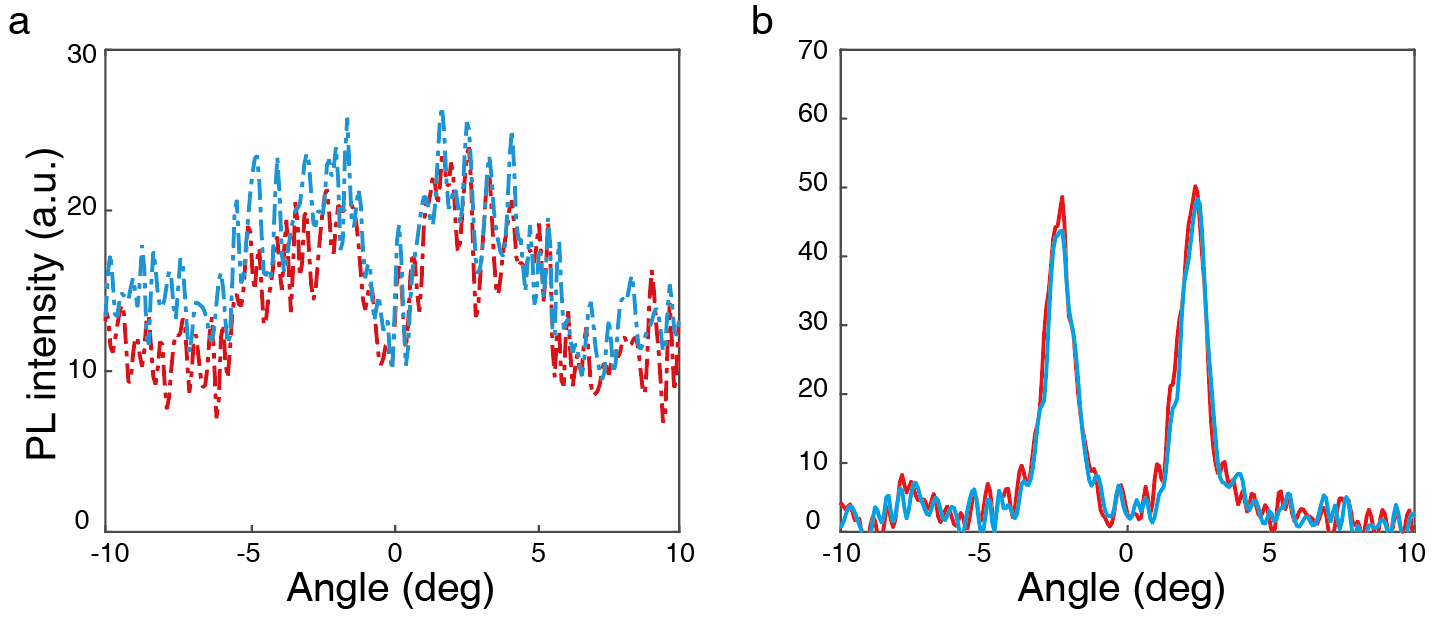


Figure S5: The red (blue) color corresponds to σ_+_ (σ_-_) polarized light. a-b The PL spectra of WS_2_ monolayer on substrate with in-plane inversion symmetry at 615 nm (a) and 628 nm (b). The σ_+_ and σ_-_ PL maximums overlap in the same angle. It shows the σ_+_ and σ_-_ photons didn’t separate in the momentum space when the WS_2_ monolayer was placed on substrate with in-plane inversion symmetry.

**7. Directional PL enhancement in the far field**


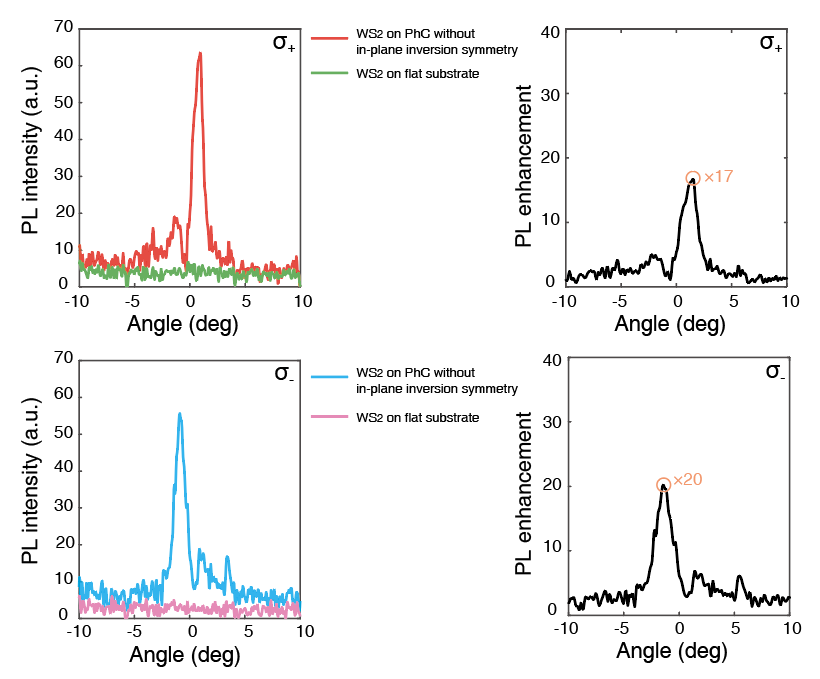


Figure S6: **Angle-resolved PL spectra at 628 nm.** The data correspond to Fig. 3a-b, e-f of the manuscript.

The PL enhancement here is calculated by dividing PL intensity of WS_2_ on the PhC by the average PL intensity of WS_2_ on flat substrate. The angle-solved PL intensity of WS_2_ on flat substrate is weak and almost uniform in this small angle range. Therefore, we used the average PL intensity to replace the exact PL intensity of WS_2_ on flat substrate. The results show a maximum of 20-fold directional σ_-_ PL enhancement at 628 nm.

**8. Calculated degree of valley polarization**


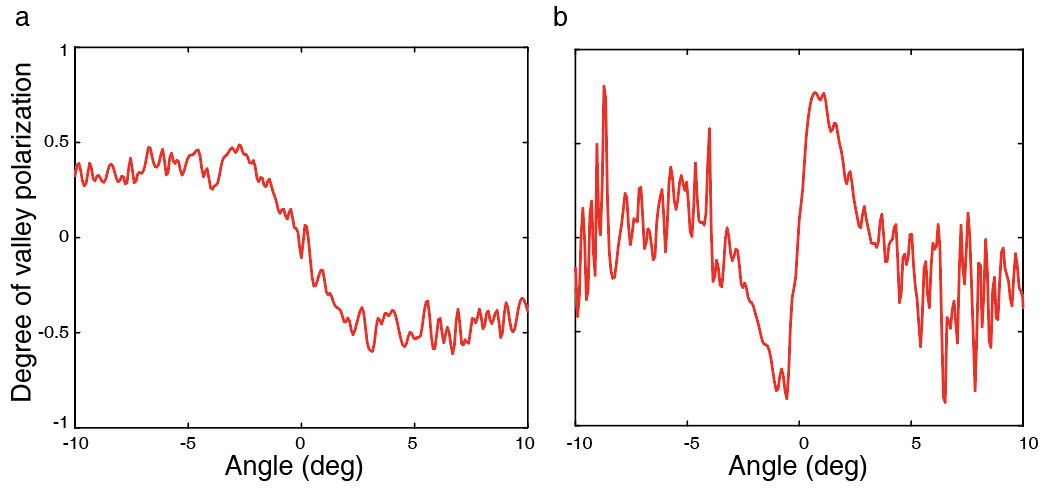


Figure S7: **Calculated degree of valley polarization.** a-b correspond to PL spectra in Fig. 3g-h. As defined in the article, the degree of valley polarization is calculated for PL at 615 nm (a) and 628 nm (b). As we can see in Fig. 3h of the article, the total intensity of σ_+_ and σ_-_ PL is nearly zero in large-angle regions, leading to strong fluctuations. We just need to concentrate on small-angle regions where the total intensity of σ + and σ − is large. The maximum valley polarization is up to 84%.

**9. Simulated transmittance along high-symmetry directions and Iso-frequency contours**


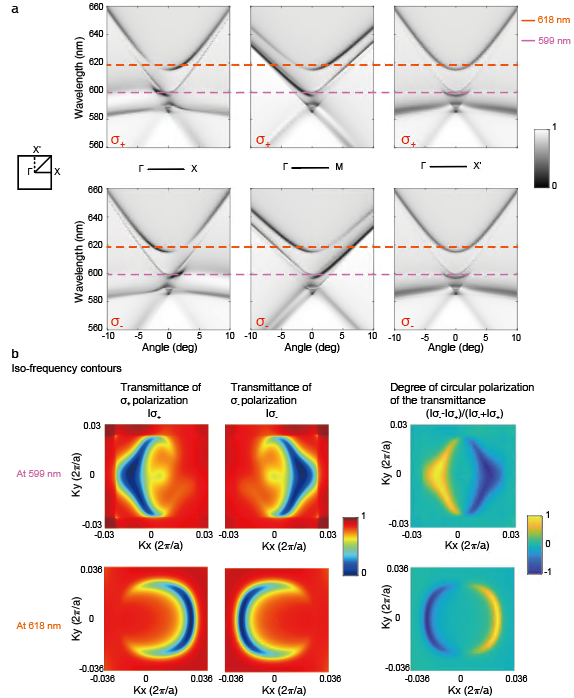


Figure S8: **Simulated transmittance along high-symmetry directions and Iso-frequency contours.** a. Transmittance spectra with σ_+_ and σ_-_ polarized incidence along Γ-X, Γ-M, and Γ-X’ directions. b. Iso-frequency contours at 599 nm and 618 nm, marked with dashed line in Fig. R2a. All these simulations are conducted by RCWA.

Figure S8a presents simulated angle-resolved transmittance spectra under circularly polarized incidence along Γ-X, Γ-M, and Γ-X’ directions by RCWA. For transmittance spectra along Γ-X and Γ-M directions, we observe that the spectra are asymmetric under circularly polarized incidence, which correspond to optical states with different circular polarization. In contrast, transmittance spectra along Γ-X’ direction are symmetric and the same as each other under σ_-_ and σ_+_ polarized incidence. This result is as expected, for the momentum space is mirror-symmetric with respect to Γ-X’ direction, and the optical states along Γ-X’ direction are symmetric-protected to be linearly polarized.

Moreover, by simulating the iso-frequency contours, we shows simulated transmittance spectra along all directions. Figure S8b illustrates the iso-frequency contours under σ_-_ and σ_+_ polarized incidence and the calculated degree of circular polarization at 599 nm and 618 nm. The results are accordant with Fig. 4 of the manuscript. And the differences between simulations and experimental measurements come from deviations of real samples’ fabrications and optical parameters.

**10. Time-resolved PL measurement under linearly polarized pulsed laser excitation**


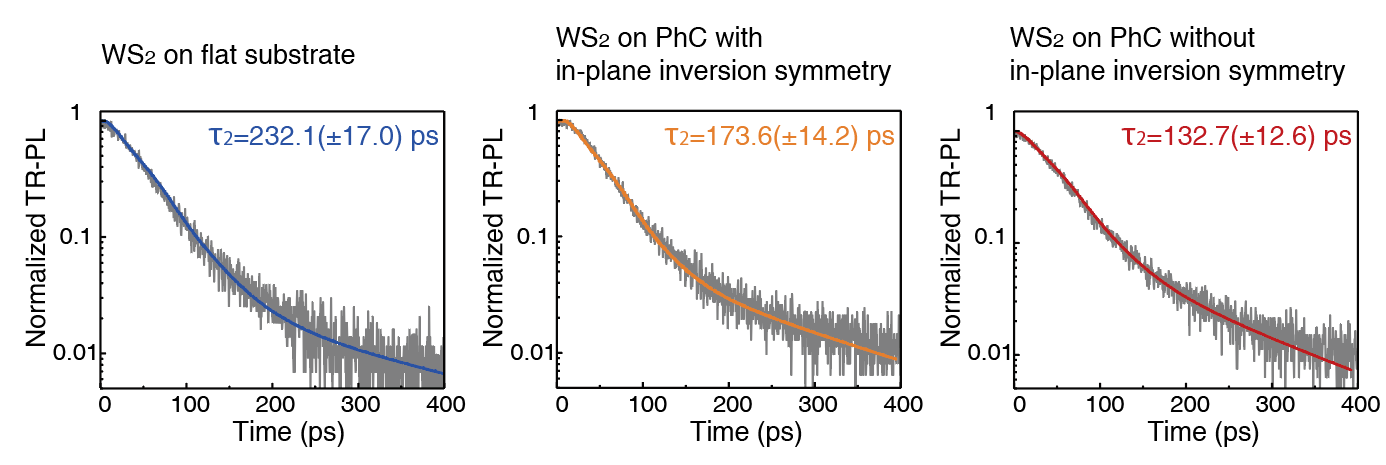


Figure S9: **Time-resolved PL measurement under linearly polarized pulsed laser excitation.** The colored solid lines are the fitting curves of the time-resolved PL measurements.

| Substrate of the monolayer WS_2_ | Non-radiative | | **Radiative** | |
| --- | --- | --- | --- | --- |
|  | A (%) | $\tau_{1}$ (ps) | 1-A (%) | $\boldsymbol{\tau}_{\boldsymbol{2}}$ **(ps)** |
| PhC slab without in-plane inversion symmetry | 91.8 | 32.6±6.3 | 8.2 | **132.7±12.6** |
| PhC slab with in-plane inversion symmetry | 93.7 | 29.4±5.8 | 6.3 | **173.6±14.2** |
| Flat substrate | 95.9 | 36.8±6.8 | 4.1 | **232.1±17.0** |

Table S1: **Fitting results for time-resolved PL measurements.** The TR-PL signal is modelled as $I\left( t \right)=A\cdot e^{-\frac{t}{\tau_{1}}}+(1-A)\cdot e^{-\frac{t}{\tau_{2}}}$, where A (1-A) and τ_1_ (τ_2_) represent the ratio and lifetime of modelled non-radiative (radiative) process, respectively^7^.

In this TR-PL experiment, both WS_2_ on designed PhC slabs and flat substrate were excited under 400 nm laser of 100 fs pulse-width and corresponding TR-PL decay traces were collected by a time-correlated single photon counting (TCSPC) device. The experimental setup is provided in **section 4**.

**11. Optical photos of monolayer WS_2_ on different substrates**


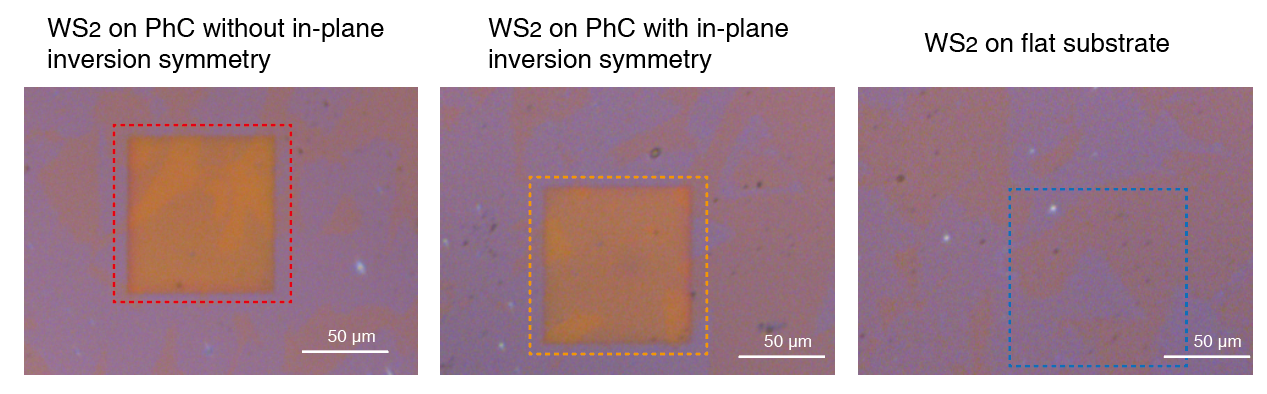


Figure S10: **Optical photos of monolayer WS_2_ on different substrates.** The first two photos show WS_2_ on PhC slabs without and with in-plane inversion symmetry, and the last one shows the WS_2_ on flab substrate.

**12. Near-filed simulations and experimental PL images for the selective coupling of circularly polarized modes in the in-plane inversion-symmetry broken PhC slab**


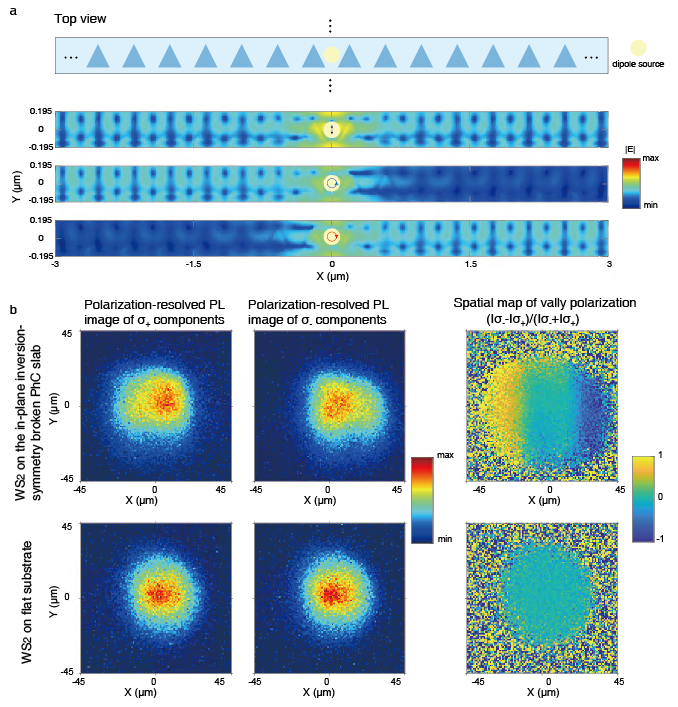


Figure S11: a. Simulated electric field intensity distribution induced by linear, σ_-_ and σ_+_ dipoles on the in-plane inversion-symmetry broken PhC slab. The simulations are conducted by FDTD Solutions. b. Polarization-resolved PL images of σ_+_ and σ_-_ components and spatial map of valley polarization.

We performed near-filed simulations to reveal how circularly polarized dipole emitters with different helicity can excite different PhC resonances, as is presented in Figure S11a. The simulations are based on the finite-difference time-domain method. And we notice that there is a slight wavelength shift of band structures compared with simulations by RCWA for different simulation methods. The monitored wavelength is 636 nm which is near the circularly polarized states under the simulated parameters. We show the top view of a row of the structures along Γ-X direction, with the source marked with a pale-yellow round in the center. Periodic boundary condition is applied on the y-direction boundaries. Simulated electric field intensity distributions centered at the Si_3_N_4_ layer with linear, σ_-_ and σ_+_ dipole sources are presented in order. The near field profile is symmetric with a linear dipole source. In contrast, the near field profiles induced by σ_-_ and σ_+_ dipoles are asymmetric and σ_-_ and σ_+_ light are guided to opposite in-plane propagating directions, with σ_-_ for left and σ_+_ for right in this simulation. In conclusion, we confirmed that circularly polarized dipole emitters with different helicity can selectively excite different PhC resonances.

Moreover, we measured polarization-resolved PL images by a lab-built PL system, as presented in Figure S11b. Here, we used a visible bandpass filter (620 nm, FWHM 10 nm, Thorlabs FB620-10) in this measurement. For WS_2_ on the in-plane inversion-symmetry broken PhC slab, the PL images of σ_+_ and σ_-_ components are asymmetric, demonstrating the asymmetric propagation of polarized PL in the PhC slab. Then we calculated and mapped the valley polarization in the real space as below, indicating valleys’ separation in the real space. For comparison, PL images and valley-polarization map of WS_2_ on flat substrate show no asymmetric propagation and valleys’ separation in the real space.

**13. Degree of valley polarization in momentum space for WS_2_ monolayer on flat substrate**


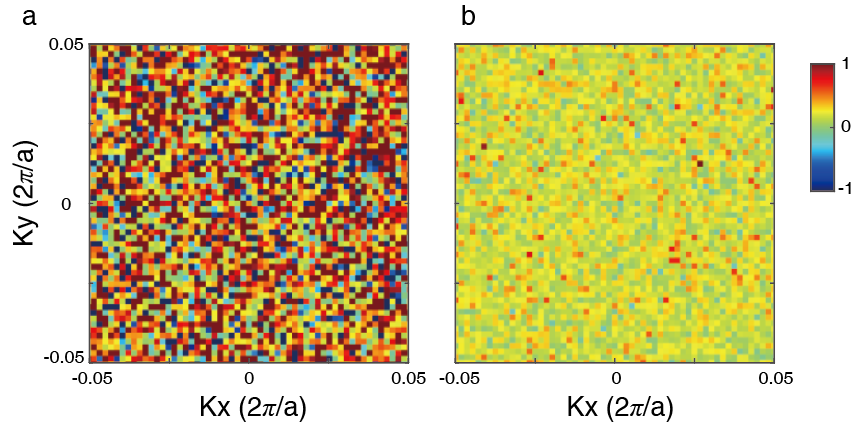


Figure S12: **Images of valley polarization P(k) in momentum space at 605 nm (a) and 628 nm (b).** When the WS_2_ monolayer is placed on s flat substrate, the PL intensity is weak, leading to strong fluctuations. The PL intensity at 628 nm is relatively large, so the fluctuations are smaller. We can see there are not evident areas with same signs of P(k), which means valley photons don’t separate in the momentum space.

**14.** **The scanning electron microscopy image of the double-slit**


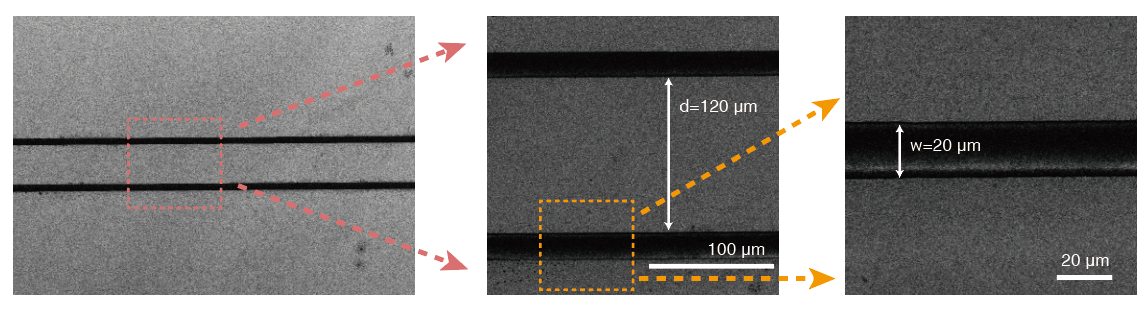


Figure S13: **The scanning electron microscopy image of the double-slit.** The double-slit distance d is 120 microns. Each slit has a 20-micron width.

**15.** **Angle-resolved spectra without circularly-polarized detection**


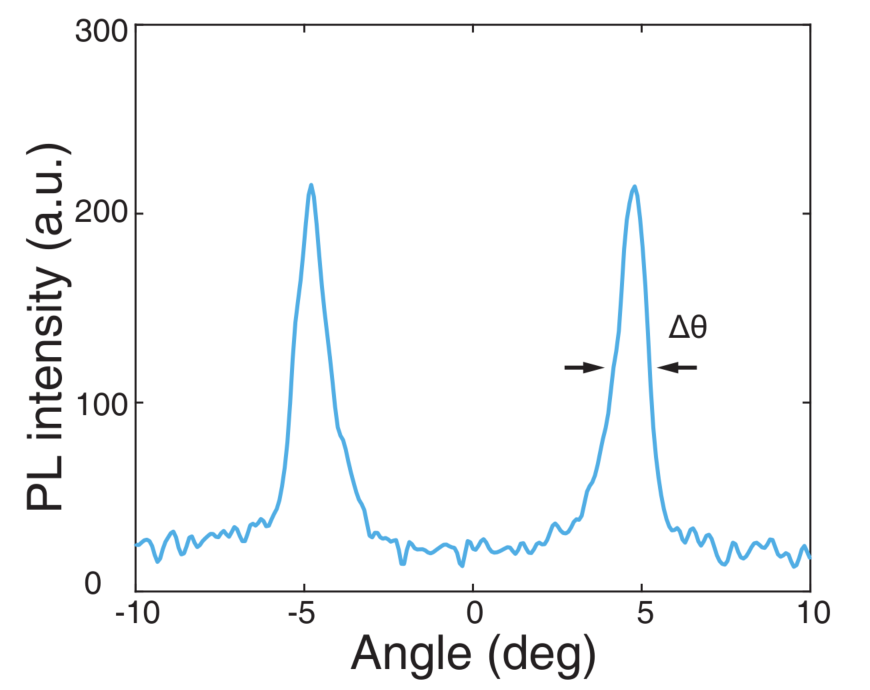


Figure S14: **Angle-resolved spectra without circularly-polarized detection at 621 nm.** We just move QWP 2 and Linear polarizer 2 out of the measurement system to detect the angle-resolved PL spectra. The full width at half maximum of PL peaks (∆θ) is 1.23°.

**16. Simulated angle-resolved transmittance spectra of PhC with up-down mirror symmetry about the centric sample plane**


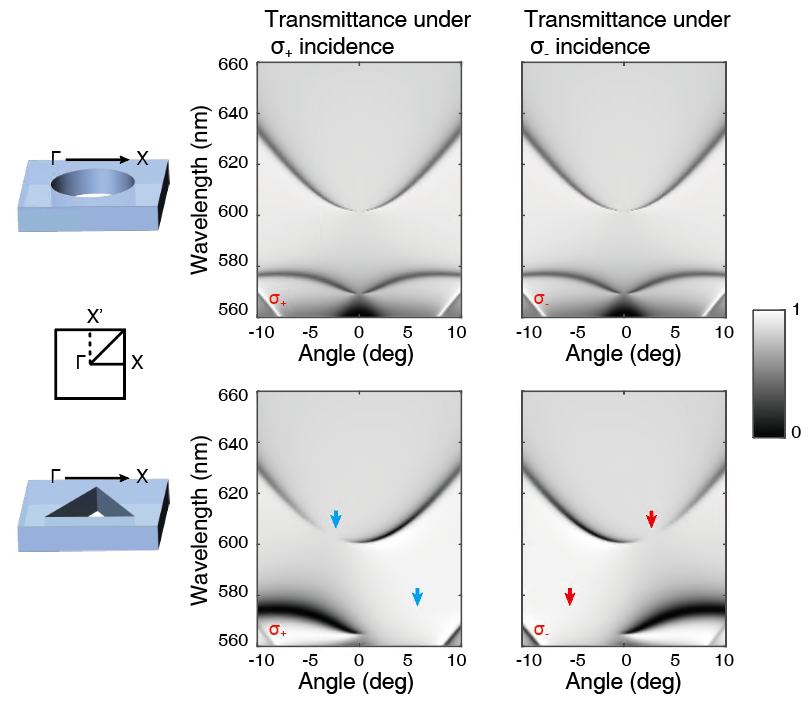


Figure S15: Simulated angle-resolved transmittance spectra of PhC with up-down mirror symmetry about the centric sample plane. The upper (lower) row corresponds to simulations of PhC slab with (without) in-plane inversion symmetry. More details about parameters are shown in supplementary materials.

The broken symmetry in the z-plane due to the substrate is not necessary to generate circularly polarized states. To show this, we simulated the cases of PhC with up-down mirror symmetry about the centric sample plane, as presented in Figure S14. We can see that by breaking the in-plane inversion symmetry, circularly polarized states emerge, which is similar to Fig. 2. In our work, samples with a substrate are used to easy the WS_2_ transfer process.

**17. References**

[1] Carminati, R. & Greffet, J.-J. Near-field effects in spatial coherence of thermal sources. Phys. Rev. Lett. 82, 1660 (1999).

[2] Shi, L. et al. Coherent fluorescence emission by using hybrid photonic–plasmonic crystals. Laser Photonics Rev. 8, 717–725 (2014).

[3] Fort, E. & Grésillon, S. Surface enhanced fluorescence. J. Phys. Appl. Phys. 41, 013001 (2007).

[4] Kwon, M.-K. et al. Surface-plasmon-enhanced light-emitting diodes. Adv. Mater. 20, 1253–1257 (2008).

[5] Rivera, P. et al. Valley-polarized exciton dynamics in a 2D semiconductor heterostructure. Science 351, 688–691 (2016).

[6] Zhang, Y. et al. Observation of polarization vortices in momentum space. Phys. Rev. Lett. 120, 186103 (2018).

[7] Palummo, M., Bernardi, M. & Grossman, J. C. Exciton radiative lifetimes in two-dimensional transition metal dichalcogenides. Nano Lett. 15, 2794–2800 (2015).
